# mascRNA and its parent lncRNA MALAT1 promote proliferation and metastasis of hepatocellular carcinoma cells by activating ERK/MAPK signaling pathway

**DOI:** 10.1038/s41420-021-00497-x

**Published:** 2021-05-17

**Authors:** Shu-Juan Xie, Li-Ting Diao, Nan Cai, Li-Ting Zhang, Sha Xiang, Chang-Chang Jia, Dong-Bo Qiu, Chang Liu, Yu-Jia Sun, Hang Lei, Ya-Rui Hou, Shuang Tao, Yan-Xia Hu, Zhen-Dong Xiao, Qi Zhang

**Affiliations:** 1 Vaccine Research Institute of Sun Yat-sen University, The Third Affiliated Hospital of Sun Yat-sen University, Guangzhou, 510630 China; 2 Biotherapy Center, The Third Affiliated Hospital of Sun Yat-sen University, Guangzhou, 510630 China; 3 Department of Hepatic Surgery and Liver Transplantation Center, The Third Affiliated Hospital of Sun Yat-sen University, Guangzhou, 510630 China

**Keywords:** Long non-coding RNAs, Cancer epigenetics

## Abstract

MALAT1-associated small cytoplasmic RNA (mascRNA) is a cytoplasmic tRNA-like small RNA derived from nucleus-located long noncoding RNA (lncRNA) metastasis-associated lung adenocarcinoma transcript 1 (MALAT1). While MALAT1 was extensively studied and was found to function in multiple cellular processes, including tumorigenesis and tumor progression, the role of mascRNA was largely unknown. Here we show that mascRNA is upregulated in multiple cancer cell lines and hepatocellular carcinoma (HCC) clinical samples. Using HCC cells as model, we found that mascRNA and its parent lncRNA MALAT1 can both promote cell proliferation, migration, and invasion in vitro. Correspondingly, both of them can enhance the tumor growth in mice subcutaneous tumor model and can promote metastasis by tail intravenous injection of HCC cells. Furthermore, we revealed that mascRNA and MALAT1 can both activate ERK/MAPK signaling pathway, which regulates metastasis-related genes and may contribute to the aggressive phenotype of HCC cells. Our results indicate a coordination in function and mechanism of mascRNA and MALAT1 during development and progress of HCC, and provide a paradigm for deciphering tRNA-like structures and their parent transcripts in mammalian cells.

## Introduction

The majority of human genome is transcribed and engenders a complex network of transcripts including ~20,000 protein coding RNAs and hundreds of thousands of transcripts with little or no protein coding capacity^1,2^. Besides lots of these noncoding RNAs function as regulators of gene expression, a significant fraction of them can serve as precursors for small RNAs^3^. For example, H19, a 2 kb lncRNA, has been shown to serve as microRNA precursor^4^. pre-tRNAs or mature tRNAs have been found to produce tRNA-derived fragments (tRFs), which represent a new category of regulatory noncoding RNAs with distinct biological functions in cancers and stress-induced diseases^5,6^. Also, a recent study computationally identified more than 100 RNAs possessing unusual 3′ end structure, suggesting a unique processing mechanism to generate small RNAs from long transcripts^7^.

The metastasis-associated lung adenocarcinoma transcript 1(MALAT1) is one of the most well-known lncRNAs^8^. Unlike most other lncRNAs that are poorly conserved across genomes, MALAT1 is highly conserved in vertebrates^7,9^. Interestingly, processing MALAT1 nascent transcript yields a long nuclear-retained ncRNA and a cytoplasmic small tRNA-like RNA derived from 3′ end^7,10,11^. The generation of MALAT1-associated small cytoplasmic RNA (mascRNA) mainly contains two steps: RNase P cleaves the nascent MALAT1 transcript at its 3′-end, downstream of a poly(A)-rich sequences, to generate the 5′ end of mascRNA; the tRNA-like mascRNA is further cleaved by RNase Z to generate a 58nt product, and then a CCA motif was added to its 3′ end by CCA-adding enzyme to yield a 61nt matured mascRNA^10^. mascRNA folds to a tRNA structure and thus was recognized by canonical tRNA processing machinery include RNase P, RNase Z, and CCA-adding enzyme. However, it does not contain a conserved anticodon loop and is not aminoacylated, mascRNA is probably not involved in transferring a specific amino acid during protein synthesis^10^. Interestingly, by analysis of the secondary and tertiary structure of the 3′ end of MALAT1 RNA, a family of lncRNAs with a unique 3′ end structure similar to that of MALAT1 were identified in vertebrate, suggesting a novel class of lncRNAs with a conserved gene structure and posttranscriptional processing^7^.

Plenty of studies are focused on MALAT1^12,13^. Upregulation of MALAT1 has been observed in various cancers^13^, including breast cancer^14^, lung cancer^8^, bladder cancer^15^, colorectal cancer^16^, etc. Several groups have reported that MALAT1 could function as an oncogene by promoting cell proliferation and increasing cell migration in lung cancer^17^, cervical cancer^18^, colorectal cancer^19^, and breast cancer^14^. Since MALAT1 is ubiquitously expressed in almost all human tissues and upregulated in almost all types of cancer^12,13^, further studies of MALAT1 in other cancers, such as HCC, will undoubtedly provide insight into its roles in tumorigenesis.

However, functional studies on mascRNA are very limited as yet. There was one report that mascRNA was involved in cardiovascular innate immunity, but the underlying mechanism is unknown^20^. Additionally, MALAT1 transcripts with several kb locate in nucleus, whereas mascRNAs with dozens of bp locate in cytoplasm^10^. Whether mascRNA has functions in cancers, and whether these two different resided transcripts coordinate cellular process have not been answered.

In this study, we found that mascRNA was highly expressed in multiple cancer cell lines, and was correspondingly upregulated in HCC clinical samples. Overexpression of mascRNA greatly promoted HCC cell proliferation and metastasis both in vitro and in vivo. Its parent RNA, MALAT1, was also confirmed as an oncogene in HCC by inhibiting cell proliferation and metastasis when it was knocked down. Furthermore, we surprisingly found that these two RNAs can both target ERK/MAPK signaling pathway, suggesting a coordinated function and mechanism between MALAT1 and mascRNA in HCC cells.

## Results

### mascRNA is highly expressed in multiple cancer cell lines and HCC clinical samples

**Fig. 1:**
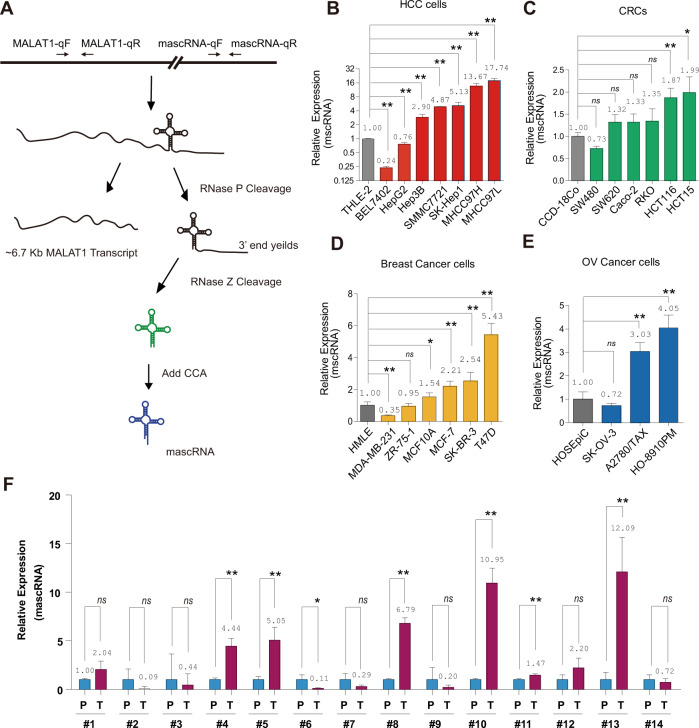
mascRNA is enriched in multiple cancer cell lines and HCC clinical samples. **A** Schematic diagram of mascRNA biogenesis. The position of primers for real-time RT-PCR of mature mascRNA or MALAT1 were marked with arrows on the upper diagram. mascRNA expression levels were measured by real-time RT-PCR in (**B**) normal hepatocyte THLE-2 and seven liver cancer cell lines (BEL7402, HepG2, Hep3B, SMMC-7721, SK-Hep1, MHCC97H, and MHCC97L), **C** normal colorectal cell line CCD-18Co and six colorectal cancer cell lines (SW480, SW620, Caco-2, RKO, HCT116, and HCT15), **D** HMLE and six breast cancer cell lines (MDA-MB-231, ZR-75-1, MCF10A, MCF-7, SK-BR-3, and T47D), **E** HOSEpiC and three ovarian cancer cell lines (SK-OV-3, A2780/TAX, and HO-8910PM), respectively. **F** mascRNA expression levels in 14 human primary HCC tissue (T) and paracancer tissue (P). Quantitative data are represented as mean ± SD. *P < 0.05, **P < 0.01.

mascRNA is processed from 3′-end of lncRNA MALAT1 as shown in Fig. 1A. Given that the sequences of matured mascRNA and MALAT1 transcript do not overlap, their expression levels could be steadily distinguished by real-time RT-PCR with different primers (Fig. 1A). We profiled their expression patterns in liver cancer, colorectal cancer, breast cancer, and ovarian cancer cell lines, respectively. As shown in Fig. 1B, among seven liver cancer cell lines, mascRNA was significantly upregulated in five HCC cell lines, including MHCC97L, MHCC97H, SK-Hep1, SMMC-7721, and Hep3B, comparing with a normal liver cell line THLE-2. mascRNA also has significantly higher expression levels in HCT15 and HCT116 colorectal cancer cell lines as comparing with normal colorectal cell line CCD-18Co (Fig. 1C). In six breast cancer cell lines, the expressions of mascRNA in T47D, SK-BR-3, MCF-7, and MCF10A were higher than that of HMLE (Fig. 1D). We also found mascRNAs’ expressions were upregulated in two ovarian cancer cell lines (HO-8910PM and A2780/TAX) (Fig. 1E). All these results demonstrated that mascRNA is highly expressed in multiple cancer cell lines. However, its parent RNA, MALAT1, is partially upregulated in some cancer cell lines (Fig. S1), which might attribute to the limited cancer cells.

To gain further insight into mascRNA expression in tumors, 14 pairs of HCC clinical samples were collected. Compared to paracancerous tissues, mascRNA was significantly upregulated in six samples as assayed by real-time RT-PCR (Fig. 1F), implying it might play a oncogenic role in HCC. Meanwhile, we also profile the expression of MALAT1 in these samples, and MALAT1 was found significantly upregulated in most samples (Fig. S2). In majority of cases, MALAT1 and mascRNA have similar expression patterns, indicating that the expression of mascRNA coincided with that of MALAT1 in HCC samples, and suggesting that mascRNA and MALAT1 may have similar function in HCC.

### mascRNA promotes proliferation and invasion of HCC cells in vitro

**Fig. 2:**
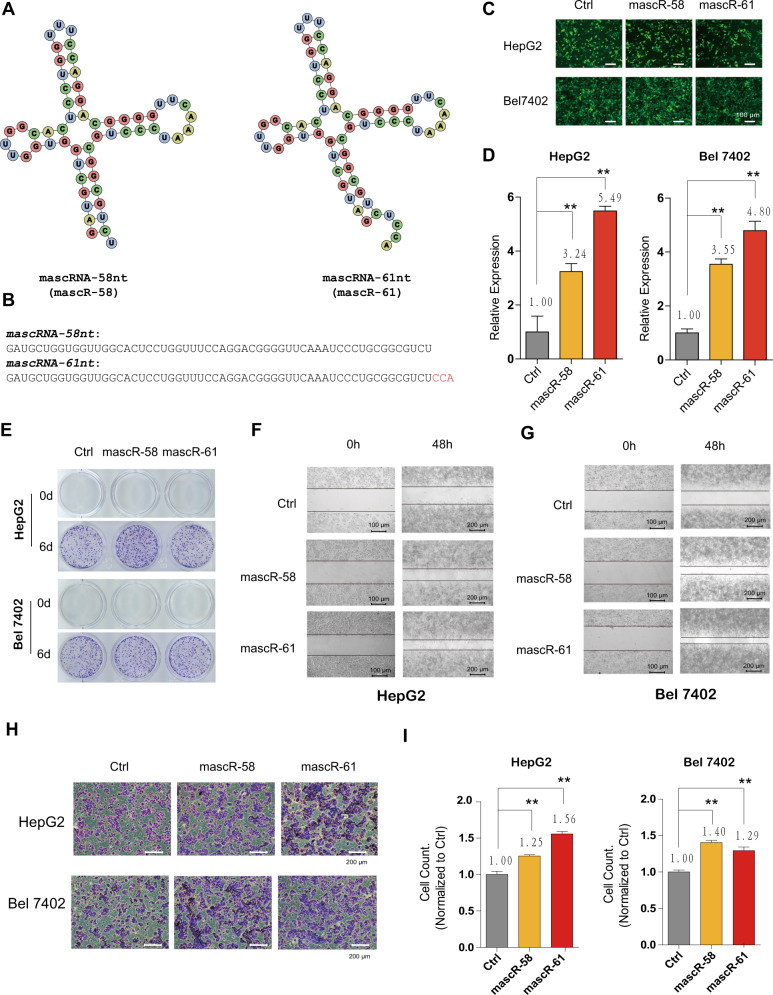
mascRNA promotes HCC cell proliferation and metastasis in vitro. **A** Secondary structure diagram of mascRNA without the terminal CCA (mascR-58, 58nt) and mature mascRNA containing CCA (mascR-61, 61nt). **B** Sequences of mascRNA-58nt and mascRNA-61nt. **C** GFP fluorescent signals of HepG2 and Bel7402 cells with control/oe-mascR-58nt/oe-mascR-61nt lentivirus infection. Scale bar represents 100 μm. **D** The expressions of mascRNA in HepG2/Bel7402 + control/oe-mascR-58nt/oe-mascR-61nt stable cells were measured by real-time RT-PCR. **E** The effect of mascRNA overexpression on cell proliferation was detected by colony formation assay. 1000 HepG2 or Bel7402 stable cells were seeded for each well in 12-well plate. Colonies were stained with 0.1% crystal violet. Wound healing assays were performed in (**F**) HepG2 and (**G**) Bel7402 stable cell lines. Movement of cells into wound was shown at 0 h (Scale bar represents 100 μm and 48 h (Scale bar represents 200 μm post scratch. Cell migration was assessed by recover of the scratch. **H** Transwell matrigel invasion assay showing the effect of mascRNA overexpression on the invasive activity of HepG2 or Bel7402 stable cells. Scale bar represents 200 μm. **I** The graphics presents the number of invaded cells, normalized to the control group. Quantitative data were represented as mean ± SD. *P < 0.05, **P < 0.01.

Since mascRNA is consistently upregulated in cancer cells and HCC clinical samples, we tried to identify the pathological function of mascRNA in HCC cells. As short in length, effective siRNAs/shRNAs targeting mascRNA are not available, thus we performed mascRNA overexpression in HepG2 and Bel7402 cells, respectively, in which the expression of mascRNA was relatively low. The mascRNA overexpression vectors were constructed in two ways: mascRNA without the terminal CCA (mascR-58, 58nt) or mature mascRNA containing CCA (mascR-61, 61nt), followed as Gast et al.^20^. The secondary structures and sequences of mascRNA with and without CCA were shown in Fig. 2A, B, respectively. The mascRNA overexpression vectors contain an exogenous GFP gene downstream mascRNA sequences. All stable cells expressed GFP showed green color in Fig. 2C, suggesting that these GFP positive cells were successfully infected by lentivirus. Then the abundance of mascRNA was measured by real-time RT-PCR. As comparing to the control cells, the expression levels of mascR-58 and mascR-61 were increased about three to six fold (Fig. 2D), suggesting mascR-58 and mascR-61 were successfully overexpressed in HepG2 and Bel7402 cells.

As shown in Fig. 2E, overexpression of either mascRNA mascR-58 or mascR-61 in HepG2 and Bel7402 cells promote cell proliferation, examined by crystal violet staining assay. Such result is consistent with a recent report which also found mascRNA can promote cell proliferation, although using different cell lines^21^. To investigate the effect of mascRNA overexpression on the migration ability of HCC cells, the scratch wound healing assay was performed. Cells were allowed to fill the gap for 48 h, and the results showed HepG2 (Fig. 2F) and Bel7402 (Fig. 2G) cells with mascRNA overexpression migrated significantly faster than the control group, suggesting that overexpression of mascRNA could greatly increase the HCC cell migration. We next determined the invasive ability of mascRNA overexpressed stable cells using transwell invasion assays. As shown in Fig. 2H, I, tumor cell invasion was significantly enhanced in HepG2 and Bel7402 stable cells overexpressing mascR-58 or mascR-61. All these results suggested that mascRNA has oncogenic effect on HCC cells by promoting cell proliferation, migration, and invasion.

### mascRNA promotes tumor growth and metastasis of HCC cells in vivo

**Fig. 3:**
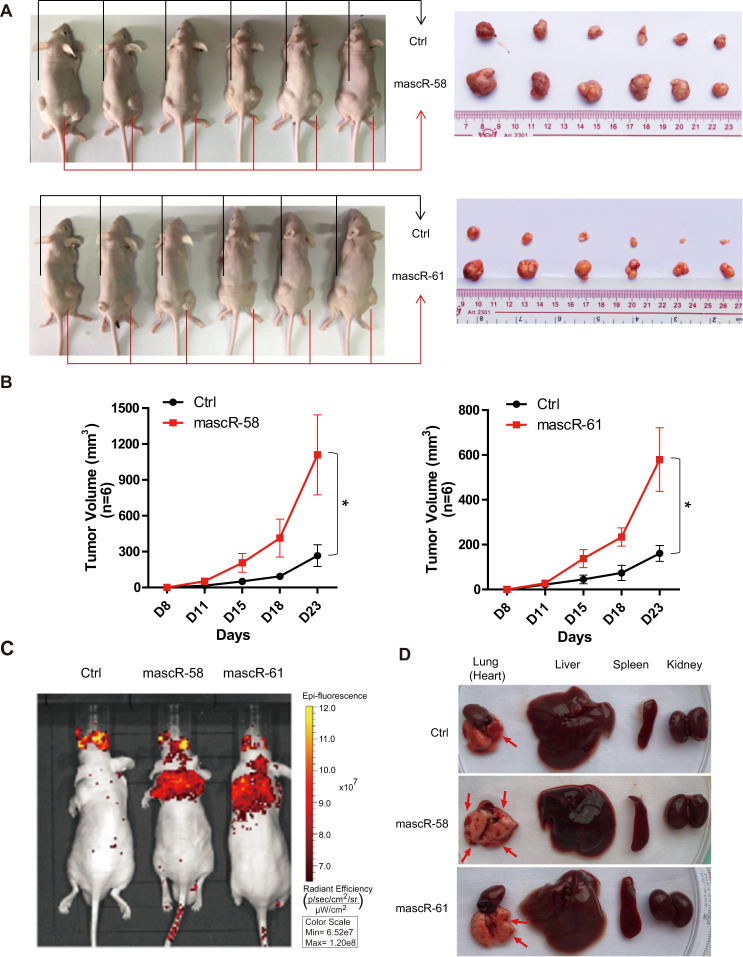
mascRNA promotes HCC tumor growth and metastasis in vivo. **A** Subcutaneous tumor formation in male BALB/c-nu mice. mascR-58nt or mascR-61nt overexpressed Bel7402 stable cells were injected subcutaneous on the right side of mice, while the same number of corresponding control cells were injected on the left side of the same mice. After 4 weeks, the mice were dissected, and the tumors grown under the skin were removed, measured, and photographed. **B** The tumor volumes were measured on day 8, 11, 15, 18, and 23 after subcutaneous injection. *n* = 6/group. **C** In vivo metastasis assay of Bel7402 stable cells was performed by tail intravenous injection. Representative images of mice with the scramble control (Ctrl), mascR-58nt and mascR-61nt overexpression constructs were taken by the in vivo animal imaging system. The bioluminescence signals indicated the location of cancer cells. **D** Mice after the bioluminescence signal detection were sacrificed and dissected. Lung (heart), liver, spleen, and kidney were excised and the surface changes of organs were observed. Lesions in lung are indicated by red arrows. Quantitative data were represented as mean ± SD. *P < 0.05.

To investigate the role of mascRNA in tumor growth and metastasis of HCC in vivo, stable Bel7402 cells overexpressing mascR-58/mascR-61 or control vector were injected into nude mice to establish a xenograft tumor and a HCC metastasis nude mouse model. In subcutaneous tumor model, cells with mascR-58/mascR-61 overexpression were injected on the right side of mice, while the control cells were injected on the left side of the same mice in order to avoid the individual differences. The tumor growth was monitored, and the tumor volume was measured on day 8, 11, 15, 18, and 23 after subcutaneous injection. The mice were dissected on day 28, and it showed the tumors generated by Bel7402 cells with mascR-58/mascR-61 overexpression grew faster and were much bigger than those generated by the control cells (Fig. 3A, B). This result indicated that mascRNA might be an oncogene that promotes tumorigenesis in HCC in vivo.

In HCC metastasis nude mouse model, control and mascR-58/mascR-61 overexpressed Bel7402 stable cells were administered through tail intravenous injection. After 4 weeks, the metastasis of Bel7402 cells was monitored by an in vivo animal imaging system, and fluorescent signals in the thoracic region and midsection were clearly observed (Fig. 3C). The results showed that mice injected with mascR-58/mascR-61 overexpressed cells obviously had more lung metastatic foci. The appearance of liver, heart, and kidney showed no obvious change, except the spleen was enlarged (Fig. 3D). This suggested that mascRNA could promote metastasis of HCC cells in vivo.

### MALAT1, mascRNA’s parent RNA, promotes proliferation and invasion of HCC cells both in vitro and in vivo

**Fig. 4:**
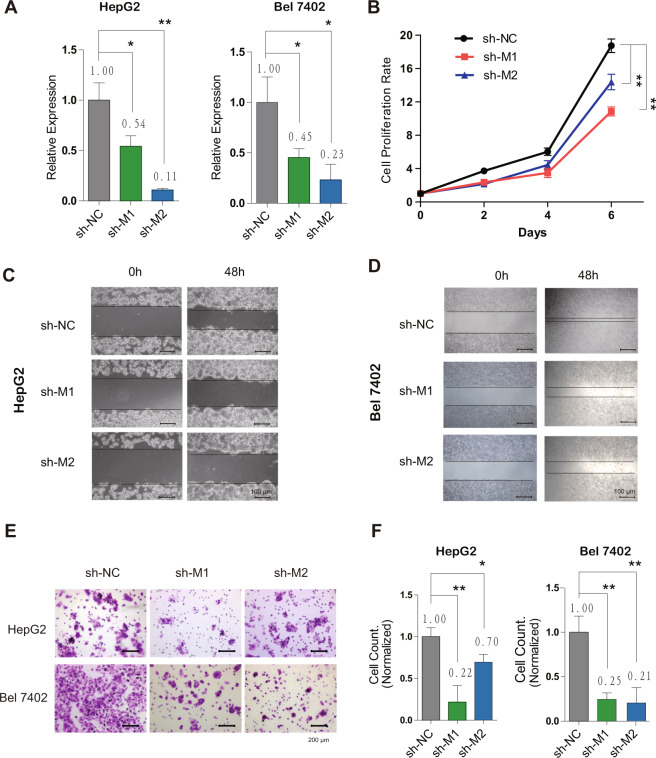
MALAT1 promotes HCC cell proliferation and metastasis in vitro. **A** The expression of MALAT1 in HepG2/Bel7402 + shNC/shMALAT1-1/shMALAT1-2 stable cell lines were measured by real-time RT-PCR. **B** CCK8 assay was performed to detect the effect of MALAT1 on cell proliferation at day 0, 2, 4, and 6. Wound healing assays were performed in (**C**) HepG2 and (**D**) Bel7402 NC/MALAT1 knockdown stable cell lines. Movement of cells into wound was shown at 0 and 48 h post scratch. Scale bar represents 100 μm. **E** Transwell matrigel invasion assay showing the effect of MALAT1 knockdown on the invasive activity of HepG2 or Bel7402 stable cells. Scale bar represents 200 μm. **F** The graphics presents the number of invaded cells, normalized to NC group. Quantitative data were represented as mean ± SD. *P < 0.05, **P < 0.01.

To investigate whether MALAT1 has the similar functions as mascRNA in HCC, we firstly knocked it down with shRNAs in HepG2 and Bel7402 cells, respectively. The sequences of shMALAT1 and shNC are listed in Table S1. The result of real-time RT-PCR showed MALAT1 was successfully downregulated (Fig. 4A), without affecting the expression of mascRNA (Fig. S3). Then the cell proliferation was examined by CCK8 assay, which showed MALAT1 downregulation could decrease HCC cell growth (Fig. 4B). In wound healing assay, HepG2 (Fig. 4C) and Bel7402 (Fig. 4D) cells with lower MALAT1 expression significantly reduced the cell migration ability. Furthermore, the result of transwell invasion assay showed a significant reduction of tumor cell invasion in MALAT1 knockdown HepG2 and Bel7402 cells (Fig. 4E), indicated by significantly less invaded cells when normalized to the control group (Fig. 4F). These results demonstrated that MALAT1 knockdown might play a protective role in HCC by decreasing cell proliferation, migration, and invasion.

**Fig. 5:**
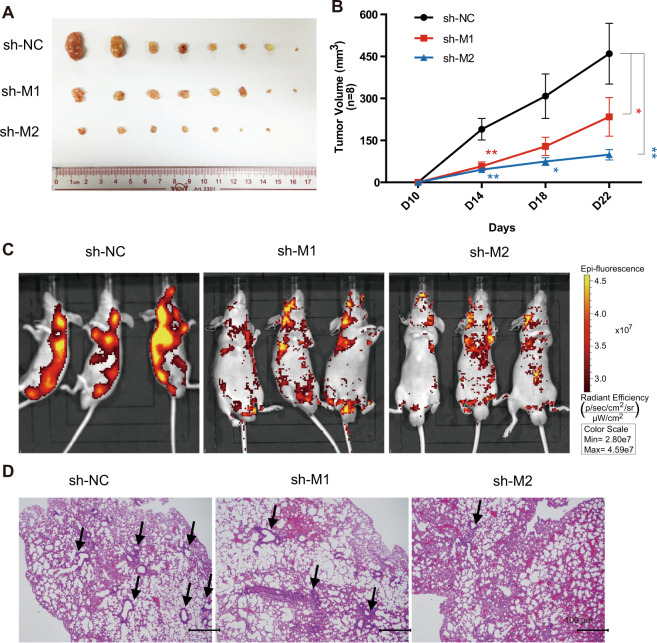
MALAT1 promotes HCC tumor growth and metastasis in vivo. **A** Subcutaneous tumor formation in male BALB/c-nu mice. shMALAT1-1 or shMALAT1-2 Bel7402 stable cells were injected subcutaneous on the right side of mice, while the same number of corresponding control cells were injected on the left side of the same mice. After 4 weeks, the mice were dissected, and the tumors grown under the skin were removed, measured, and photographed. **B** The tumor volumes were measured on day 10, 14, 18, and 22 after subcutaneous injection. *n* = 8/group. **C** In vivo metastasis assay of Bel7402 cells with MALAT1 downregulation was performed by tail intravenous injection. Representative images of mice with the scramble control (shNC), shMALAT1-1, or shMALAT1-2 knockdown constructs were taken by the in vivo animal imaging system. The bioluminescence signals indicated the location of cancer cells. **D** Mice after the bioluminescence signal detection were sacrificed and dissected. Organs were excised and the metastasis in lung was further investigated by immunohistochemistry analysis. Representative H&E staining images showing lung metastasis in control and shMALAT1 mice. Lesions in lung are indicated by black arrows. Scale bar represents 100 μm. Quantitative data were represented as mean ± SD. *P < 0.05, **P < 0.01.

In vivo experiments including subcutaneous tumor formation assay and tail intravenous injection were performed to investigate the effects on tumor growth and metastasis when MALAT1 was downregulated. As shown in Fig. 5A, when MALAT1 was knocked down, the tumor growth was seriously affected, and the tumor volume was much smaller than that of control group (Fig. 5B). MALAT1 downregulation also significantly reduced cancer metastasis of Bel7402 cells (Fig. 5C). Hematoxylin and Eosin staining showed that knockdown of MALAT1 could greatly reduce metastatic lesions in lung (Fig. 5D).

Taken together, inhibition of MALAT1 showed converse phenotypes observed with mascRNA overexpression both in vitro and in vivo, suggesting that lncRNA MALAT1 and its derived mascRNA have similar functions in promoting HCC.

### mascRNA and MALAT1 activate ERK/MAPK signaling pathway in HCC cells

The ERK/MAPK pathway is one of the most important signal transduction pathways in tumorigenesis and progression. Sorafenib, the only approved drug for HCC, inhibited tumor angiogenesis and induced tumor cell apoptosis by blocking the RAF/MEK/ERK pathway^22^, suggesting that MAPK signaling pathways, especially ERK/MAPK pathway, might play an important role in HCC. In previous studies, MALAT1 was found to affect proliferation and metastasis in various cancers via MAPK signaling pathways^23–32^. All these findings implied that MALAT1 might promote HCC cell proliferation and metastasis by activating MAPK signaling pathway.

**Fig. 6:**
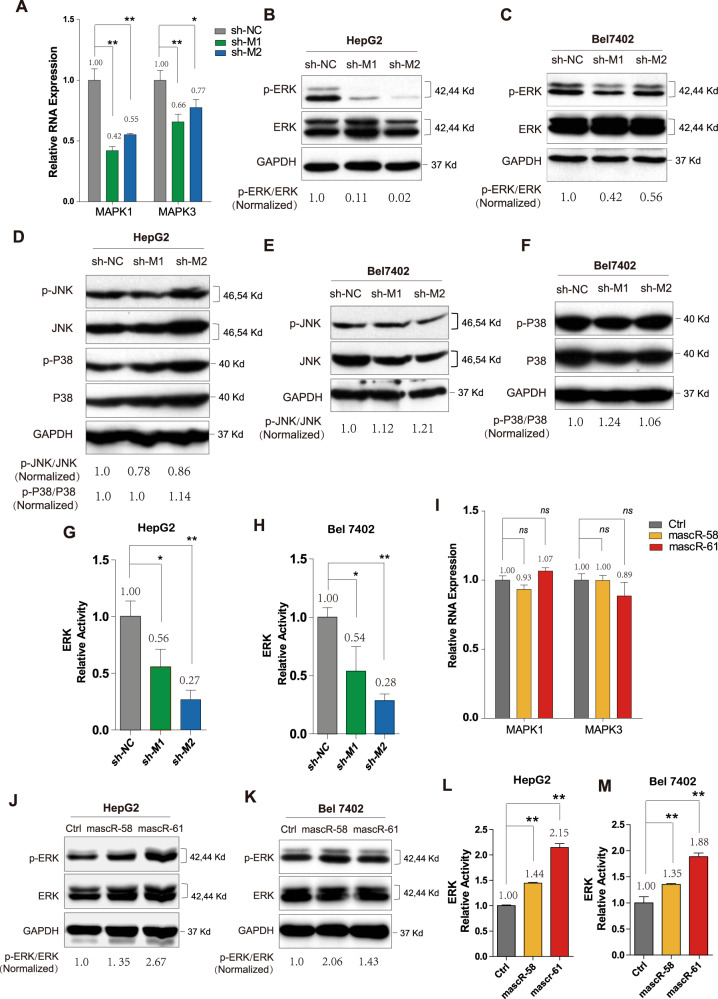
mascRNA and MALAT1 active ERK/MAPK signaling pathway in HCC. **A** The expressions of MAPK1 and MAPK3 were detected by real-time RT-PCR in control and shMALAT1 HepG2 stable cell lines. Western blotting for ERK and p-ERK in (**B**) HepG2 and (**C**) Bel7402 control and shMALAT1 stable cells. **D** Western blotting for JNK, p-JNK, P38, p-P38 in HepG2 control and shMALAT1 stable cells. Western blotting for (**E**) JNK, p-JNK, **F** P38, p-P38 in Bel7402 control and shMALAT1 stable cells. The activity of ERK signaling was examined by the dual-luciferase reporter assay in (**G**) HepG2 and (**H**) Bel7402 control and shMALAT1 stable cells. **I** The expressions of MAPK1 and MAPK3 were detected by real-time RT-PCR in control and mascRNA overexpressed HepG2 stable cell lines. Western blotting for ERK and p-ERK in (**J**) HepG2 and (**K**) Bel7402 control and mascRNA overexpressed stable cells. The activity of ERK signaling was examined by the dual-luciferase reporter assay in (**L**) HepG2 and (**M**) Bel7402 control and mascRNA overexpressed stable cells. All western blotting in this figure used GAPDH as a control. The band density of ERK, p-ERK, JNK, p-JNK, P38, and p-P38 were normalized to respective GAPDH, and the values of p-ERK/ERK, p-JNK/JNK, and p-P38/P38 were showed in the figure. Quantitative data were represented as mean ± SD. *P < 0.05, **P < 0.01.

To test our hypothesis, we firstly examined the mRNA expressions of MAPK1 and MAPK3, which were involved in ERK/MAPK pathway (Figs. 6A and S4). It showed that MAPK1 and MAPK3 were significantly downregulated when MALAT1 was knocked down, suggesting that ERK/MAPK pathway might be affected by MALAT1. Then we detected the phosphor-ERK (p-ERK) and total ERK protein levels by western blot in HepG2 and Bel7402 MALAT1 stable knockdown cells, respectively. And the results showed the levels of p-ERK were dramatically decreased (Fig. 6B, C). We also detected the phosphor-JNK (p-JNK) and phosphor-P38 (p-P38) expressions, and found neither of them significantly changed (Fig. 6D–F). Furthermore, the activity of ERK/MAPK pathway was measured by dual-luciferase reporter assay, using shNC as control. The luciferase signals were dramatically decreased when MALAT1 was knockdown (Fig. 6G, H). All these data suggested that MALAT1 might affect MAPK pathways mainly through ERK signaling in HCC cells.

We next detected the activity of ERK/MAPK pathway in mascR-58/mascR-61 overexpressed HepG2 and Bel7402 stable cells. Although the mRNA levels of MAPK1 and MAPK3 were not affected (Figs. 6I and S5), the p-ERK protein level was indeed increased when mascRNA was overexpressed in HepG2 and Bel7402 cells (Fig. 6J, K). The increased activity of ERK signaling was also confirmed by dual-luciferase reporter assays (Fig. 6L, M). Since mascRNA activated ERK signaling without affecting the mRNA expressions of MAPK1 and MAPK3, it might affect p-ERK at post-transcription level.

### mascRNA and MALAT1 regulate metastasis genes through ERK/MAPK signaling

**Fig. 7:**
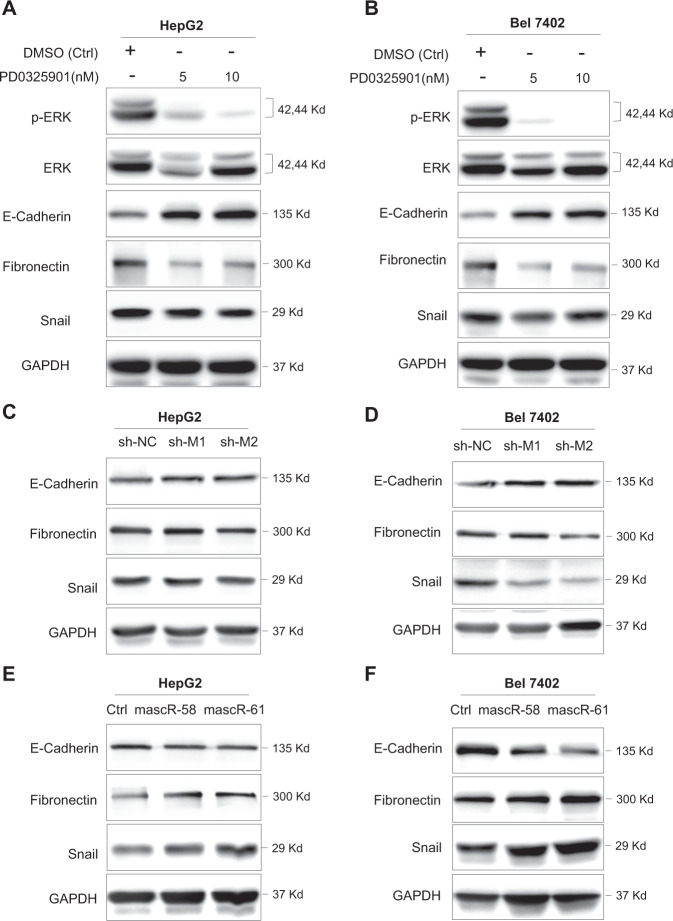
mascRNA and MALAT1 regulate metastasis genes through ERK/MAPK signaling. Western blotting for p-ERK, ERK, E-Cadherin, Fibronectin, Snail in (**A**) HepG2 and (**B**) Bel7402 cells treated with DMSO or 5/10 nM PD0325901. Western blotting for E-Cadherin, Fibronectin, Snail in (**C**) HepG2 and (**D**) Bel7402 control and shMALAT1 stable cells. Western blotting for E-Cadherin, Fibronectin, Snail in (**E**) HepG2 and (**F**) Bel7402 control and mascRNA overexpressed stable cells. GAPDH was used as control.

To further figure out the connection between ERK signaling pathway and cell metastasis in HCC, we used PD0325901, a MEK/ERK pathway specific inhibitor, to inhibit the activity of ERK/MAPK pathway in HepG2 and Bel7402 cells, respectively. As shown in Fig. 7A, B, the ERK signaling was successfully inhibited, which was indicated by the dramatic decrease of p-ERK. Then we examined the expressions of metastasis-related marker genes. E-cadherin was significantly elevated when cells were treated with PD0325901, while Snail and Fibronectin were decreased (Fig. 7A, B), suggesting that inhibiting ERK/MAPK signaling could regulate critical metastasis genes.

Since knockdown MALAT1 in HCC cells repress ERK activity, we also examined whether MALAT1 can regulate these metastasis-related genes. When MALAT1 was knocked down in HepG2 and Bel7402 cells, E-cadherin was elevated while Snail and Fibronectin were decreased (Fig. 7C, D). This result indicated that MALAT1 knockdown and ERK pathway inhibition led to the similar effects on metastasis-related genes, suggesting that MALAT1 may affect metastasis of HCC cells by regulating ERK/MAPK signaling pathway.

We further examined the expression of the same set of metastasis-related genes in mascRNA overexpressed HepG2 and Bel7402 stable cells, and found the level of E-cadherin was downregulated while Snail and Fibronectin were upregulated when mascRNA was overexpressed (Fig. 7E, F). The effects of mascRNA overexpression on these genes are converse to the results when cells were treated with PD0325901 or shMALAT1, suggesting that mascRNA may also regulate HCC metastasis through ERK/MAPK signaling pathway.

Taken together, mascRNA and MALAT1 can both regulate the same set of metastasis-related genes, which are probably via activating ERK/MAPK pathway and thus contribute to the aggressive phenotype of HCC cells.

## Discussion

**Fig. 8:**
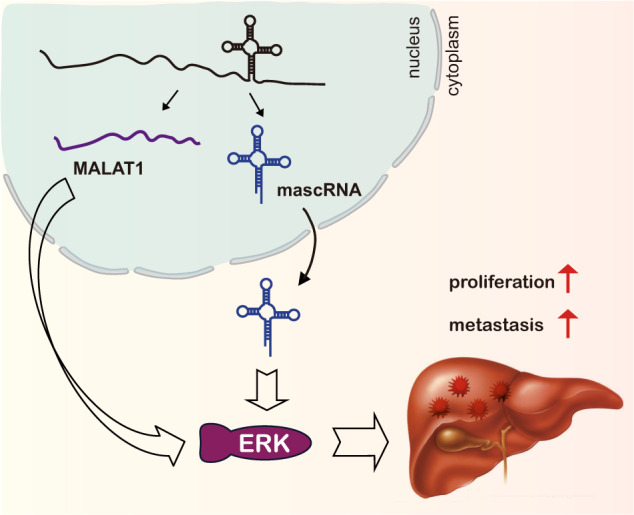
mascRNA and its parent lncRNA MALAT1 can both promote cell proliferation and metastasis of HCC cells by activating ERK/MAPK signaling. Schematic overview of mascRNA and MALAT1 in HCC.

mascRNA as the processing product of lncRNA MALAT1 was discovered more than 10 years ago^10^. Although MALAT1 has been extensively studied, the exact function of its derived mascRNA was largely unclear^12,13^. Using HCC cells as models, we showed that both mascRNA and MALAT1 can promote cell proliferation, migration, and invasion in vitro and in vivo. Furthermore, we found they can both activate ERK/MAPK signaling, and both can decrease E-cadherin while increase Snail and Fibronectin. Our results suggest an interesting function and mechanism paradigm, which two noncoding RNAs originated from the same transcript have similar functions and regulate the same signaling pathways (Fig. 8).

Due to its abundant expression and high evolutionary conservation in mammals, MALAT1 is one of the most extensive studied lncRNAs. A growing number of studies showed that MALAT1 played an important role in the proliferation and metastasis of various cancers^33–40^. Our results are consistent with several recent studies of MALAT1 in HCC, confirming that MALAT1 plays an oncogenic role in HCC by promoting cell proliferation, migration, and invasion^41–44^. Other studies also found that MALAT1 can regulate apoptosis, autophagy, and inflammatory response of HCC cells, implying that MALAT1 may coordinate multiple aspects of cell features to promote HCC progress^45,46^. In our study, mascRNA was found to have similar roles as its parent RNA MALAT1. It will be interesting to test whether mascRNA could also play roles in apoptosis, autophagy, and inflammatory response of HCC cells.

mascRNA is a tRNA-like small RNA. The stability of bona fide tRNAs is monitored by the CCA-adding enzyme. CCA is added to and thus stabilize tRNA, whereas addition of the second CCA initiates its degradation^47^. In this research, we overexpressed two forms of mascRNA, a 58nt transcript without CCA tail (mascR-58) and a 61nt transcript with CAA tail (mascR-61). Interestingly, our results showed that both mascR-58 and mascR-61 promote proliferation and metastasis of HCC cells in vitro and in vivo, suggesting that CCA tail is dispensable for mascRNA.

ERK is a key molecule transporting extracellular signaling from cell surface to nuclei^48,49^. Activation of ERK/MAPK signaling is central for cancer growth, survival, and motility of HCC cells^50,51^. It is not surprising that ERK/MAPK signaling is tightly regulated by multiple mechanisms. Besides protein regulators, previous studies revealed that typical lncRNA MALAT1 also contribute to the regulation of activity of ERK/MAPK signaling through multiple mechanisms^23–32,52^. Our study confirmed that ERK/MAPK signaling is regulated by MALAT1 in HCC cells. Interestingly, we found that mascRNA derived from MALAT1 can also regulate the activity of ERK/MAPK signaling. MALAT1 is mainly located in the nucleus, where it localizes to nuclear speckles and paraspeckles. Whereas mascRNA is found to be distributed in the cytoplasm^10^. As their locations are completely different, it is unlikely that they could directly target and regulate the same gene, which in turn modulate the activity of ERK/MAPK signaling. Such conclusion is supported by our observation that MALAT1 could affect the mRNA levels of MAPK1 and MAPK3 and thus regulate the activity of ERK/MAPK pathway, whereas mascRNA only affect the activity of ERK/MAPK signaling without regulating the mRNA levels of MAPK1 and MAPK3. It will be great interesting to elucidate the underlined molecular mechanism of both MALAT1 and mascRNA, to reveal how these two noncoding RNAs regulate the same targeting pathway, ERK signaling.

Overall, in the present study, we showed that both mascRNA and its parent MALAT1 can promote cell proliferation, migration, and invasion of HCC cells, and also can activate the ERK/MAPK signaling, suggesting a coordination in function and mechanism of mascRNA and MALAT1.

## Materials and methods

Detailed materials and methods are described in the Supplementary Information.

### Cell lines and RNA samples

HEK293T, Bel7402, HepG2, Hep3B, SMMC-7721, Sk-HEP-1, MHCC97H, HMCC97L, and THLE-2 cell lines were provided by Prof. Qi Zhang’s laboratory. Bel7402 cells were maintained in RPMI-1640 medium (GIBCO) supplemented with 10% heat-inactivated fetal bovine serum (FBS, GIBCO) and penicillin/streptomycin (Invitrogen, Carlsbad, CA). Other cell lines were maintained in Dulbecco’s Modified Eagle’s Medium (DMEM, GIBCO) supplemented with 10% heat-inactivated FBS and penicillin/streptomycin. Cells were cultured in 37 °C incubator with 5% CO_2_.

RNA samples of breast cancer cells, colorectal cancer cells, and ovarian cancer cells were gift from Prof. Jianyou Liao (Sun Yat-sen Memorial Hospital, Sun Yat-sen University, Guangzhou), Prof. Lianghu Qu (School of Life Sciences, Sun Yat-sen University, Guangzhou) and Dr. Youqiu Xue (The Third Affiliated Hospital of Sun Yat-sen University, Guangzhou), respectively.

### HCC clinical samples

We investigated a consecutive series of 14 patients with histologically confirmed HCC, who were admitted to The Third Affiliated Hospital of Sun Yat-sen University (Guangzhou, China) from May 2019 to July 2020. Patients were included with the following inclusion criteria as previously reported^53^. The study was approved by the Clinical Research Ethics Committee of The Third Affiliated Hospital of Sun Yat-sen University (Guangzhou, China) and was conducted in accordance with the ethical guidelines outlined in the Declaration of Helsinki. A written informed consent was obtained from all the patients at the time of admission.

### Animal studies

BALB/c athymic nude mice (BALB/c-nu, male, 4 weeks old) were purchased from Guangdong Medical Laboratory Animal Center, China. The animals were randomly assigned to different groups and were maintained in an approved animal facility under specific pathogen-free condition. They were housed four or five per stainless-steel wire cage, without bedding, under controlled temperature (18–26 °C) and humidity (50 ± 20%), and with a 12 h light/12 h dark cycle. All animals were provided a standard diet. All animal care and surgical procedures were approved by the Animal Ethics Committee at The Third Affiliated Hospital of Sun Yat-sen University.

Bel7402 stable cells (5.0 × 10^6^) were suspended in 100 μl × PBS and then injected subcutaneously into the dorsal flank of BALB/c-nu mice. And tail intravenous injection model was used for in vivo metastasis assay (1.0 × 10^6^ cells each). The mice were sacrificed about 4 weeks after injection. Tumors were measured and dissected, fixed in 4% formalin, and then embedded in paraffin.

### Statistical analysis

Data from at least three experiments were expressed as mean ± SD, unless specified otherwise. Student’s *t*-test was used to analyze the data, and a difference was considered to be statistically significant if *P* < 0.05.

## Supplementary information

Supplementary materials

Figure S1

Figure S2

Figure S3

Figure S4

Figure S5

Supplymentary_Table_S1

Supplymentary_Table_S2
