# Supplementary material for: mascRNA and its parent lncRNA MALAT1 promote proliferation and metastasis of hepatocellular carcinoma cells by activating ERK/MAPK signaling pathway
Source: Cell Death Discov. 2021 May 17;7:110. doi: 10.1038/s41420-021-00497-x (PMC8128908; doi:10.1038/s41420-021-00497-x)
Supplement: Supplementary file 1 — Supplementary materials [file 41420_2021_497_MOESM1_ESM.docx]

**Supplementary materials and methods**

**RNA extraction and real-time RT-PCR**

Total RNA was isolated from cells by TRIzol reagent (Invitrogen, Carlsbad, CA) as described by manufacturer’s instruction. The quantity and quality of the total RNA were verified with NanoDrop spectrophotometer (Thermo, Wilmington DE, USA). For quantification of non-coding RNAs and mRNAs, 1μg of total RNA was reverse transcribed using PrimeScript RT-PCR kit (TaKaRa) with 20μl reaction for RT, then cDNA was diluted 10 times and 2μl was added to 10μl reaction for PCR. GAPDH gene was used as endogenous control. Expression values of RNA were calculated using the comparative Ct method. The sequences of primers are available in Supplementary Table S2.

**Plasmid constructs and stable cell line generation**

For MALAT1 knockdown, plasmids containing two interfering sequences against human MALAT1 were constructed by Genechem (Shanghai, China). For expression of mascRNA variants and GFP reporters, the synthesized 58nt and 61nt fragments were cloned into the GV309 vector (Genechem, Shanghai, China). HEK293T cells were transfected with these lentiviral plasmids and viral packaging plasmids (psPAX2 and pMD2G) to produce lentiviral particles. Transfections were performed by Lipofectamine 3000 (Invitrogen) using plasmids DNA in Opti-MEM I (Invitrogen), as recommended by the manufacturer. The target HepG2 and Bel7402 cells were then infected with the lentiviruses in the presence of polybrene (Sigma). Puromycin (Sigma) resistant clones were selected and further confirmed by real-time RT-PCR.

**Colony formation assay**

The stably infected cells were seeded in a fresh 12-well plate (500 cells per well) and maintained in DMEM containing 10% FBS for 7 days. Colonies were fixed with methanol, stained with 0.1% crystal violet for 10 min and photographed using a digital camera. The cell counting kit-8 (CCK-8) assay was conducted according to the manufacturer’s protocols (CCK-8, Dojindo, Japan).

***In vitro* tumor invasion and migration assay**

Costar transwell inserts (8μm-pore size; Corning) in 24-well plates were coated with 45μl (5 mg/ml) of Matrigel (BD Biosciences) in cold serum-free DMEM (1:8) and dried overnight at 37℃. Cells were detached with trypsin and resuspended in serum-free DMEM medium. 200μl cell suspensions (5×10^4^ cells/ml) were added to the upper chamber and complete medium containing 20% FBS was added to the bottom chambers. Cells were allowed to be invasive for 24h. After incubation, cells on the upper part of the membrane were removed with a cotton swab and cells on the bottom surface of the membrane were fixed in methanol for 10 min, stained with 0.1% crystal violet for 15 min, and photographed under a microscope. *In vitro* cell migration ability was examined by wound healing assay.

**Dual-luciferase reporter assay**

For analysis of the activity of ERK signaling pathway, the stably infected cells were transfected with ERK pathway reporter plasmids, which contain the pathway focused transcription factor responsive Firefly luciferase construct and a constitutively expressing Renilla luciferase construct, at a 50 ng/well dose in 96-well plates after seeding 24h, using the Lipofectamine 3000 reagent (Invitrogen). Lysates were collected 48h after transfection. Luciferase assays were performed using the Dual-Luciferase Reporter Assay System (Promega). The activity of luciferase was measured by GloMax™ 96 Microplate Luminometer (Promega) and the activity of Renilla luciferase was normalized to Firefly luciferase activity.

**Western blotting**

The total cell proteins were prepared using RIPA buffer (50mM Tris-HCl, pH7.4, 150mM NaCl, 0.1% (w/v) SDS, 1% (w/v) sodium deoxycholate, 1% (w/v) Triton-X-100, 0.5mM EDTA, pH8.0, 1mM PMSF, 1×cocktail and phosphatase inhibitor (Roche)). After quantification by BCA Protein Assay kit (Thermo Scientific), the total proteins were separated by 10% SDS-PAGE and transferred to nitrocellulose membrane (GE Healthcare Life science, Germany). The primary antibodies, anti-p-ERK (#9101S), anti-ERK (#9102S), anti-p-JNK (#4671), anti-JNK (#9258), anti-p-P38 (#9211), anti-P38 (#9212), anti-E-Cadherin (#14472S), anti-Fibronectin (#26836S), anti-Snail (#3879), and anti-GAPDH (#2118) were all obtained from Cell Signaling Technology. Bands on western blotting were visualized by ECL (CST) and normalized to GAPDH.

**Figure S1.** The expressions of (A) mascRNA and (B) MALAT1 were measured by real-time RT-PCR in normal hepatocyte THLE-2 and seven liver cancer cell lines (BEL7402, HepG2, Hep3B, SMMC7721, SK-Hep1, MHCC97H, MHCC97L), normal colorectal cell line CCD-18Co and six colorectal cancer cell lines (SW480, SW620, Caco-2, RKO, HCT116, HCT15), HMLE and six breast cancer cell lines (MDA-MB-231, ZR-75-1, MCF10A, MCF-7, SK-BR-3, T47D), HOSEpiC and three ovarian cancer cell lines (SK-OV-3, A2780/TAX, HO-8910PM), respectively. Quantitative data are represented as mean ± SD. *P<0.05, **P<0.01.

**Figure S2.** Expression levels of (A) mascRNA and (B) MALAT1 in 14 human primary hepatocellular carcinoma tissue (T) and para-cancer tissue (P). Quantitative data are represented as mean ± SD. *P<0.05, **P<0.01.

**Figure S3.** Real-time RT-PCR of mascRNA in HepG2 / Bel7402 control and MALAT1 knockdown stable cells. Quantitative data are represented as mean ± SD.

**Figure S4.** Real-time RT-PCR of MAPK1 and MAPK3 in Bel7402 control and MALAT1 knockdown stable cells. Quantitative data are represented as mean ± SD. *P<0.05.

**Figure S5.** Real-time RT-PCR of MAPK1 and MAPK3 in Bel7402 control and mascRNA over-expressed stable cells. Quantitative data are represented as mean ± SD.

**Table S1. Sequences of shRNAs for MALAT1 and control.**

**Table S2. Sequences of primers used in this study.**
