# Supplementary figures and images for: mascRNA and its parent lncRNA MALAT1 promote proliferation and metastasis of hepatocellular carcinoma cells by activating ERK/MAPK signaling pathway
Source: Cell Death Discov. 2021 May 17;7:110. doi: 10.1038/s41420-021-00497-x (PMC8128908; doi:10.1038/s41420-021-00497-x)

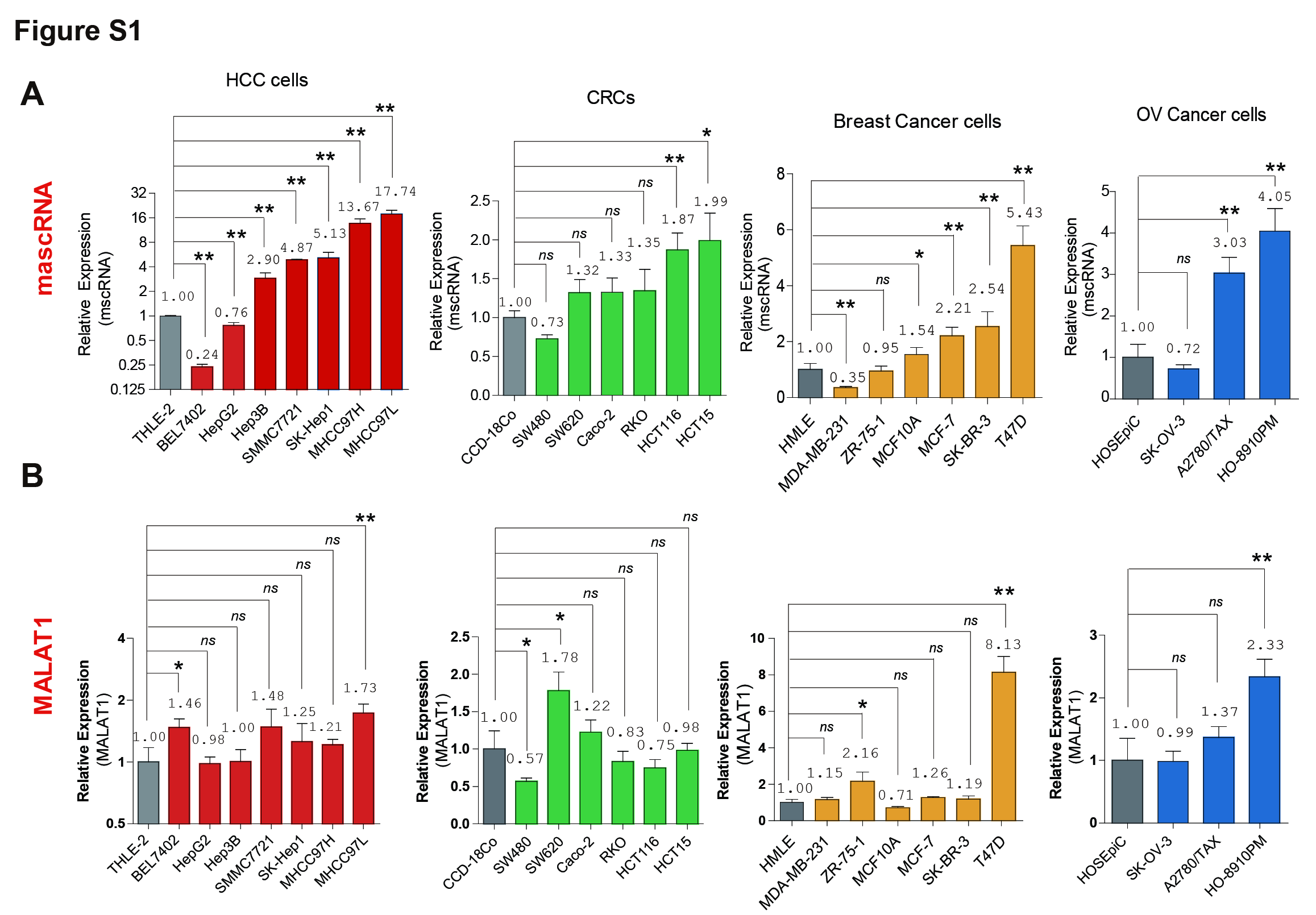

Supplement: Supplementary file 2 — Figure S1 [file 41420_2021_497_MOESM2_ESM.tif]

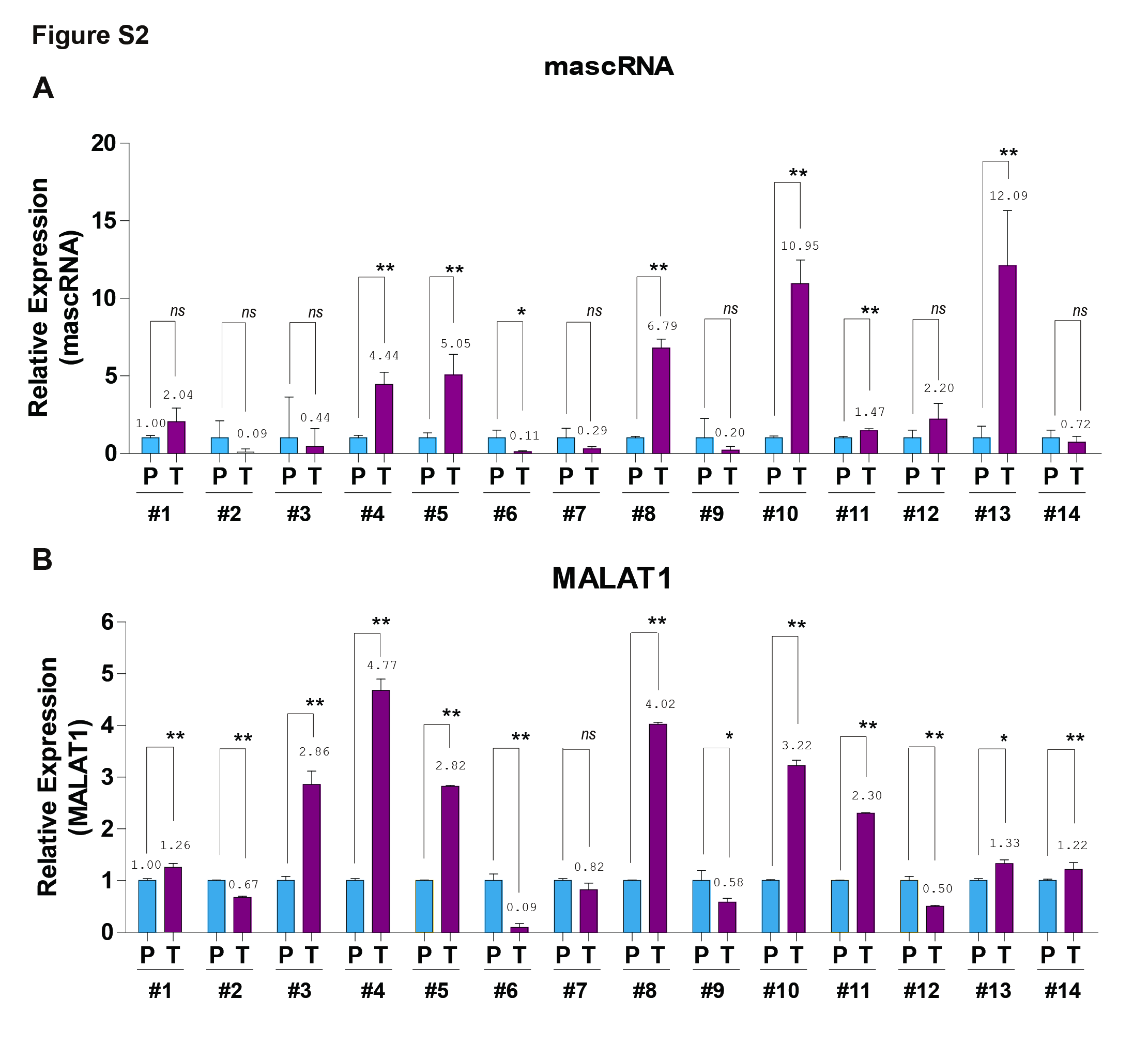

Supplement: Supplementary file 3 — Figure S2 [file 41420_2021_497_MOESM3_ESM.tif]

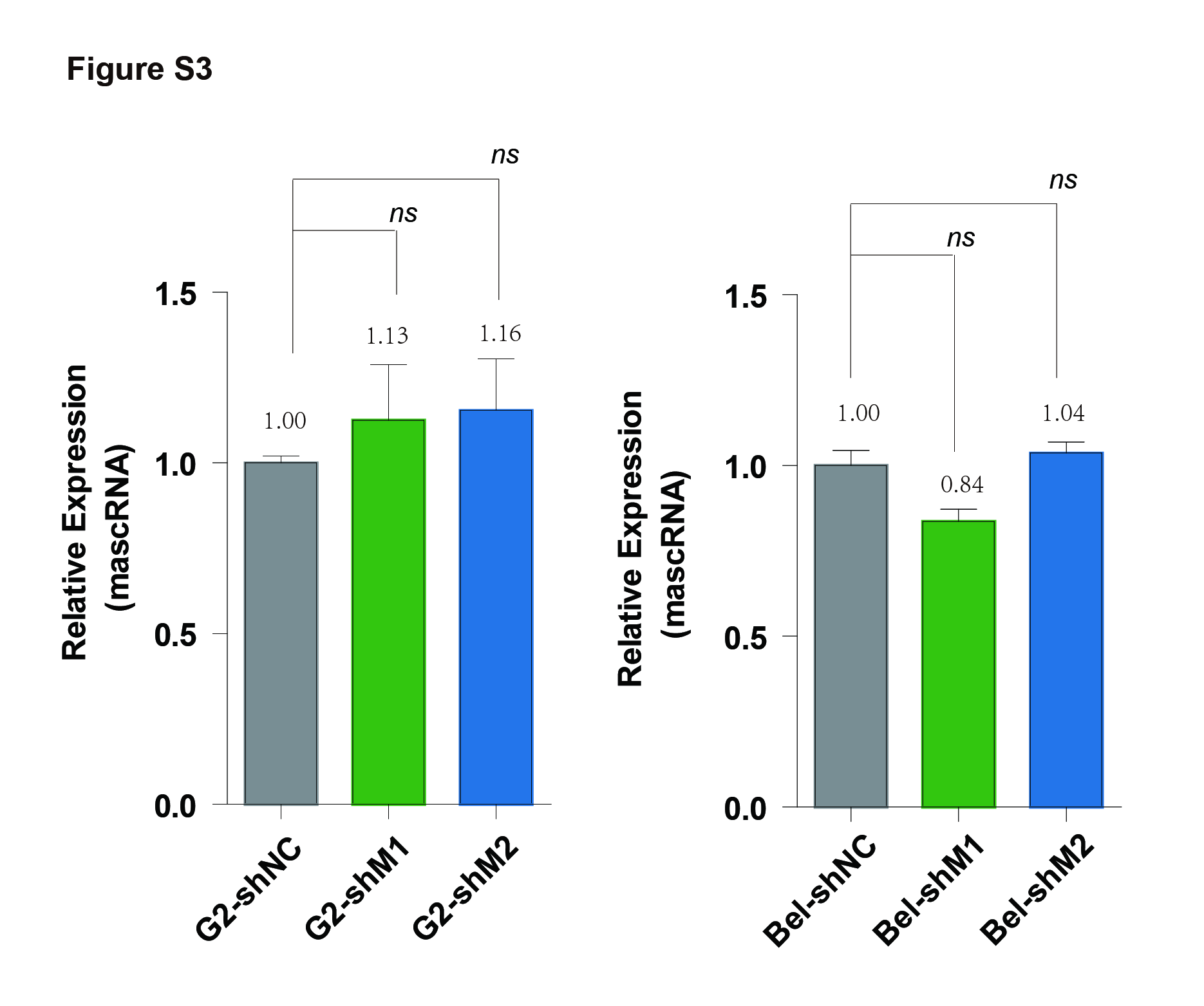

Supplement: Supplementary file 4 — Figure S3 [file 41420_2021_497_MOESM4_ESM.tif]

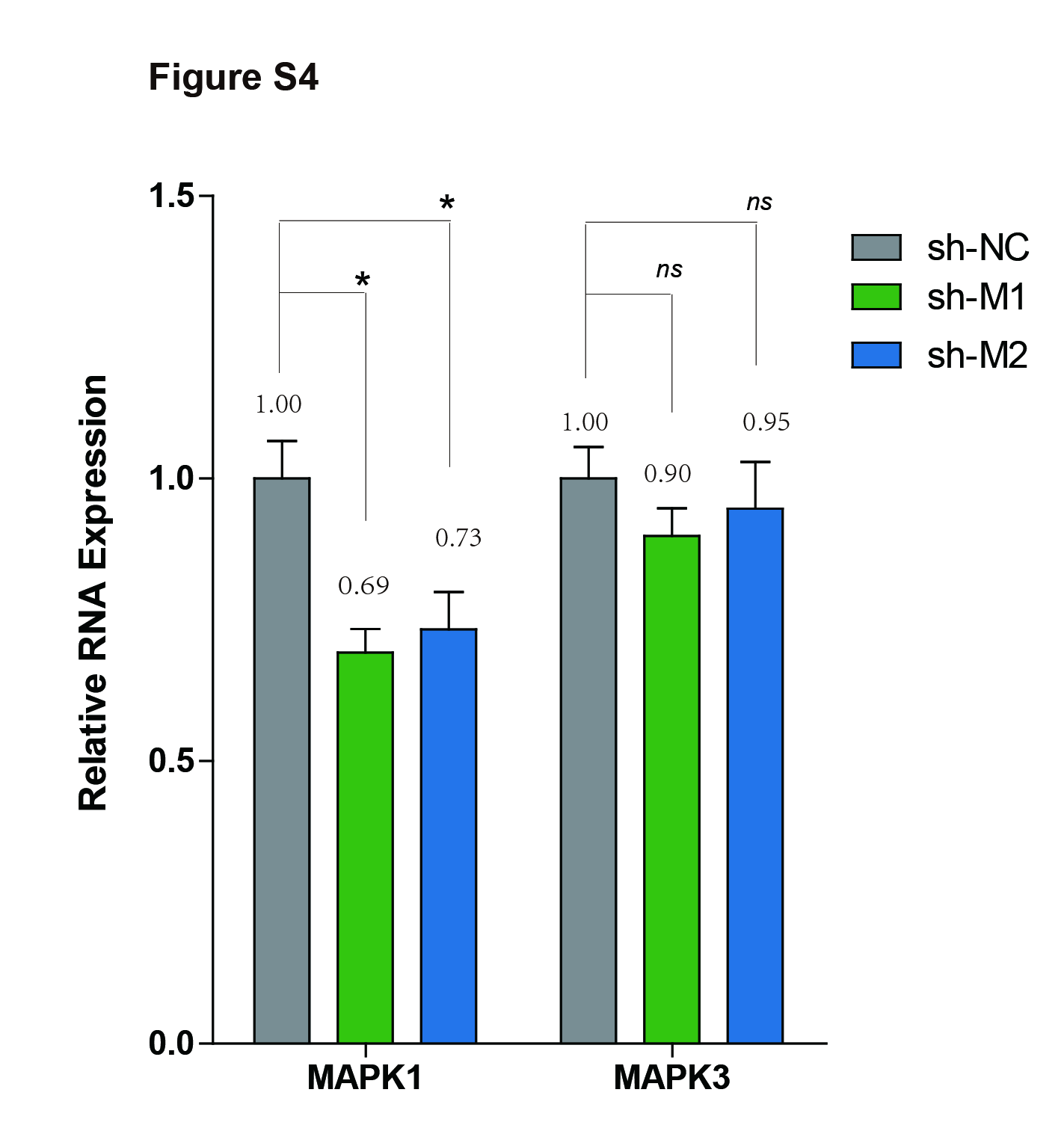

Supplement: Supplementary file 5 — Figure S4 [file 41420_2021_497_MOESM5_ESM.tif]

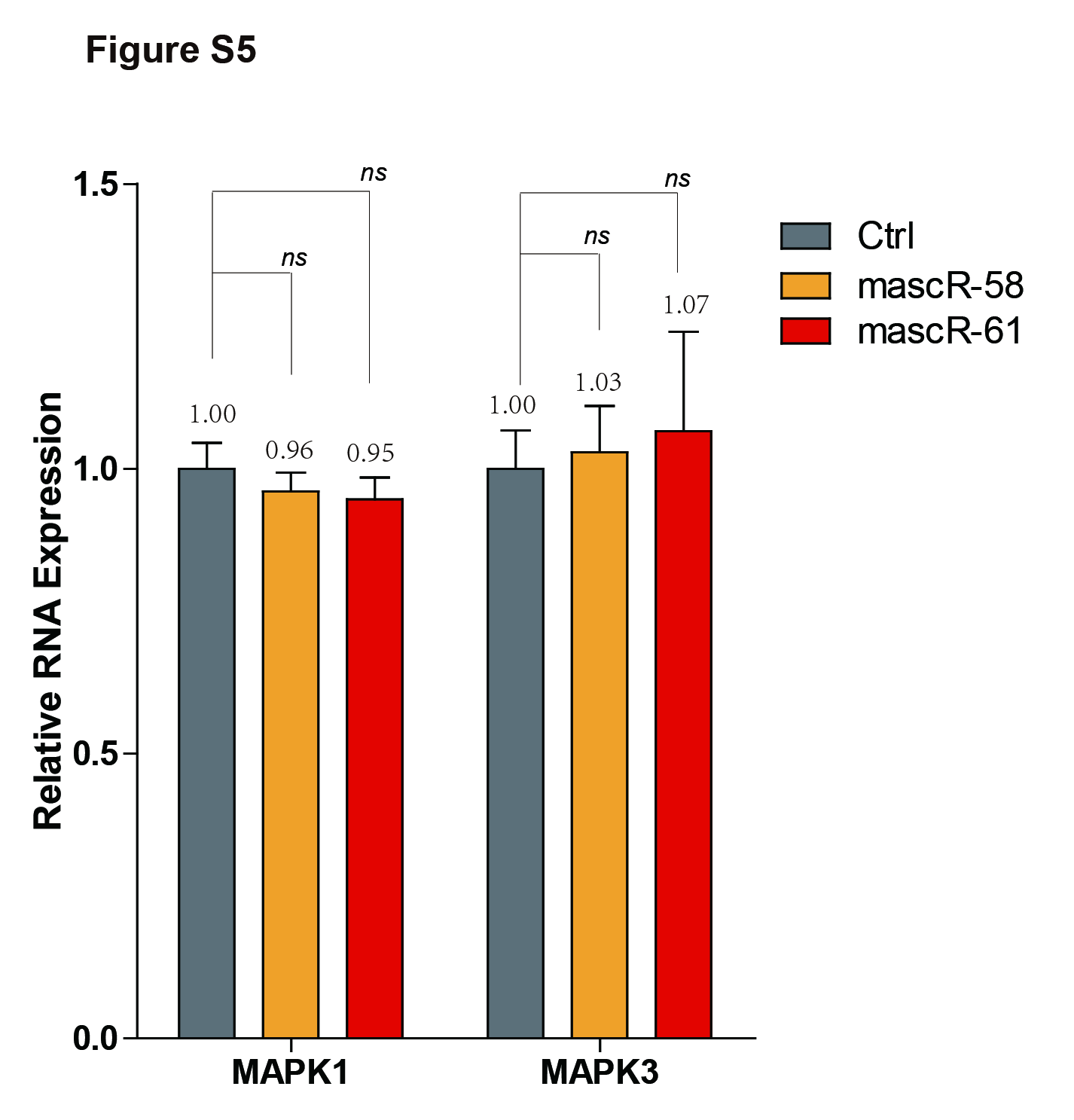

Supplement: Supplementary file 6 — Figure S5 [file 41420_2021_497_MOESM6_ESM.tif]
